# Enhanced Thermo-Optical Switching of Paraffin-Wax Composite Spots under Laser Heating

**DOI:** 10.3390/ma10050525

**Published:** 2017-05-12

**Authors:** Asmaa Said, Abeer Salah, Gamal Abdel Fattah

**Affiliations:** National Institute of Laser Enhanced Sciences, Cairo University, Cairo 12613, Egypt; soom_asmaa_a@yahoo.com (A.S.); gfattah@niles.edu.eg (G.A.F.)

**Keywords:** thermo-optical switching, paraffin wax, phase change materials, carbon fillers, graphene, graphite, composites

## Abstract

Thermo-optical switches are of particular significance in communications networks where increasingly high switching speeds are required. Phase change materials (PCMs), in particular those based on paraffin wax, provide wealth of exciting applications with unusual thermally-induced switching properties, only limited by paraffin’s rather low thermal conductivity. In this paper, the use of different carbon fillers as thermal conductivity enhancers for paraffin has been investigated, and a novel structure based on spot of paraffin wax as a thermo-optic switch is presented. Thermo-optical switching parameters are enhanced with the addition of graphite and graphene, due to the extreme thermal conductivity of the carbon fillers. Differential Scanning Calorimetry (DSC) and Scanning electron microscope (SEM) are performed on paraffin wax composites, and specific heat capacities are calculated based on DSC measurements. Thermo-optical switching based on transmission is measured as a function of the host concentration under conventional electric heating and laser heating of paraffin-carbon fillers composites. Further enhancements in thermo-optical switching parameters are studied under Nd:YAG laser heating. This novel structure can be used in future networks with huge bandwidth requirements and electric noise free remote aerial laser switching applications.

## 1. Introduction

One of the most significance advances in optical switching technology is Thermo-Optical Switching (TOS). TOS is a waveguide switching which is very attractive due to adequate speed for time critical applications and small size. The main principle of TOS is the variation of the refractive index of the material caused by temperature gradients [1,2].

Tailoring composite Phase Change Material (PCM) is of great interest mainly due to its interesting capabilities in solar energy storage, building energy savings and temperature control in electronic equipment to improve the overall system performance [3]. PCMs are linked to three energy storage methods: sensible heat, latent heat and chemical energy. Latent heat storage is an effective energy loading method due to high capacity and small temperature variation from storage to retrieval [4].

Paraffin wax is commonly used as a phase change material, exhibiting high latent heat thermal energy storage and low temperature variation, although this material suffers from low thermal conductivity ~0.24 W/m·K [3,4,5]. Graphene and graphite are recently used to enhance the low thermal conductivity of paraffin wax [6,7,8,9], and to accelerate melting and solidification heat transfer rates [10,11,12]. Graphite features excellent physical properties such as high electrical and thermal conductivities, high heat of fusion and thermal stability. Graphene has shown remarkable applicability in diverse areas as catalysis, sensors, biomedicine, composites, and energy devices [13,14,15,16].

Several high thermal conductive fillers have been reported to improve the thermal conductivity of PCMs. In this sense, various approaches have been recently reported: graphite foam [17]; compressed expanded natural graphite to improve Wood’s alloy heat transfer [18]; exfoliated graphite nanoplatelets to form paraffin composite phase change material [3]; adding various nano-additives to liquid paraffin of various sizes and shapes [16,19,20]; and adding various nanofillers such as long and short multi-walled carbon nanotubes, carbon nanofibers, graphene nanoplatelets [21] and graphene-nickel/n carboxylic acids composites [22], among many others. Other interesting routes are based in 2D ordered elements such as carbon and boron nitride, which were used by Fang et al. [23] to be incorporated in PCM with significance performance.

In this work, we address a simple thermo-optical switching technique based on the transmission change under different heating conditions by means of Nd:YAG laser as a heating source. TOS arise from the following concepts: the wax has two states, translucent (OFF) and transparent (ON), below and above the melting temperature, respectively. If a powerful heating pulse (laser pulses and electric hot filament) affects a small spot of paraffin wax, the state of the wax changes from the solid to liquid phase (OFF/ON) after certain switching time. Reports on the use of laser heating in different fields confirm the applicability of this technique, such as accelerating thermo-mechanical fatigue of metallization [24], tailor mechanical properties of steel tube [25], inducing phase change in vanadium dioxide crystals [26], and bending titanium alloy sheets [27].

This research reports on the preparation, characterization and examination of the thermo-optical switching performance of paraffin-wax spot composites under conventional electric heating and laser heating. We extend a previous work for the TOS transmission of light through paraffin wax under electric heating of 1.3 mg of wax [28,29]. Here, mille-sized spots are studied to gain a small quantity of heat in a short time undergoing to the melting phase. Hence, thermo-optical switching in spot morphology is expected to be faster. The switching temperature and the switching time are important factors for the switching performance. Thermo-optical switching has a large optical bandwidth, small physical dimensions, large fabrication tolerances, and good performances. In addition, a laser pulse is used for remote aerial switching. The switching efficiency is enhanced in the studied composites, which makes the design applicable in aerial communications.

## 2. Materials and Methods

### 2.1. Materials and Sample Preparation

The chemical preparations of the composites require the following:

- Paraffin wax from DIFCO laboratories (Detroit, MI, USA) was used as a PCM; its formula C_n_H_2n+2_ where *n* = 26. Graphite (G) and graphene (Gn) were used to enhance the thermal properties of paraffin wax. Paraffin wax, graphite and graphene have different thermal properties.
- Graphite was obtained from Johnson Matthey Catalog Company (WAR HILL, MA, USA) as a commercial graphite rod with diameter 6.15 mm and length 150 mm, and purity 99.9995%. Graphite with micron size was used in our work. A small amount of graphite powder (from) was ground from the graphite rod to prepare different concentration of graphite.
- Graphene was synthesized in Nanotech Egypt for photoelectronics. The powder graphene used in our work had black color and nano size. The synthesis of graphene was achieved by chemical reduction of graphene oxide [30,31].

Grinding the graphite or the graphene is done using agate mortar. Then, the molten wax was added to the ground particles. In the case of graphite-paraffin wax, 2.5 mL toluene was added into the molten paraffin wax to get homogenous distribution through the paraffin matrix, while no solvent was added in the case of grapheme-wax composite. Mixtures were stirred for 2 h under heating at 70 °C. Two series were prepared of paraffin wax composites: the first one is for graphite-paraffin composites dispersed in toluene with percentages 0%, 0.007%, 0.03%, 0.07%, 0.3%, 0.7%, 3% and 7%, and the second series is for grapheme-paraffin composite with percentages 0%, 0.001%, 0.005%, 0.007%, 0.03%, 0.07%, 0.3% and 0.7%. Inject a spot of paraffin wax composites (1.3 mg) in the center of capillary tube with inner diameter 1 mm in order to study the thermo optical switching properties through optic fiber light transmission.

### 2.2. Thermo-Optical Switching through Optical Fiber Transmission Setup

Thermo-optical switching under electrical heating is measured for the composites inside a glass capillary tube, while, in the case thermo-optical switching under laser heating, a copper capillary tube is used. As mentioned before, Paraffin wax composites were melted in a pot on a heating plate. Then, the molten composite spot is injected into the center of the capillary tube.

The setup components are listed below as follows:

- A laser beam from laser Max INC. (Rochester, NY, USA), output power ≤ 4.25 mw and wavelength 677 nm.
- Fused silica optical fiber with core diameter 200 µm, cladding diameter 250 µm and jacket diameter 1 mm.
- Glass capillary tube is from Micro-Hematocrites, with inner diameter 1.1–1.2 mm. The capillary tube is surrounded by helical heater with 20 watt power.
- Copper capillary tube is fabricated from copper sheet with inner diameter 1 mm.
- Thermocouple with K type, its accuracy ±1 of reading and its resolution 1 °C; the thermocouple records temperature during heating and cooling in the temperature range 20–90 °C.
- Power meter with model number THORLABS S142C: Its spectral range varies from 350 to 1100 nm and the head of power meter is Si detector.
- The Nd:YAG laser, model No. SL-10 (Continuum, Santa Clara, CA, USA), with energy pulse 110 mJ, 1064 nm wavelength, 10 Hz frequency reputation rate and 6 ns pulse duration.

**Figure 1:**
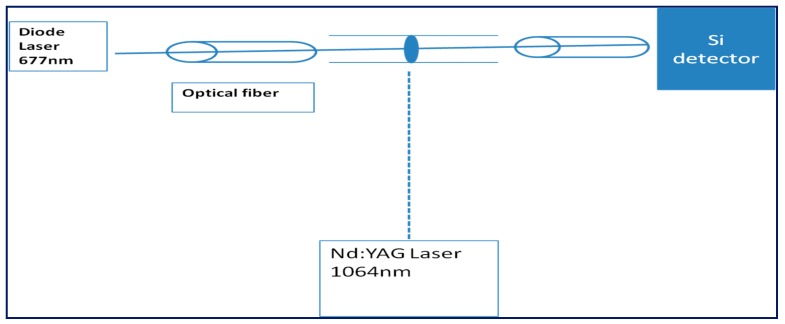
Thermo-optical switching setup using Nd:YAG as a heating source. The sample is under heating using Nd:YAG laser pulses: a laser beam 677 nm is directed to the sample in the capillary tube through optical fiber, and the transmission intensities were measured via optical power meter as a function of time.

A laser beam is directed to the sample in the capillary tube through optical fiber (Figure 1), and the transmission intensities were measured via optical power meter. The sample is under heating using laser pulses, where, for electrical heating, a helical heater is used. Using a copper sheet as a capillary tube is mandatory to bear high power of pulsed laser, as the glass capillary tube broke under exposure to laser pulses. The transmission is recorded as a function of time and temperature under heating the samples.

Thermal properties such as melting temperature, solidifying temperature, latent heat capacity, heat gained, and heat lost of paraffin wax composites were measured by differential Scanning Caliormeter (DSC-50, Shimadzu, Coulombia, MA, USA). The thermal properties of paraffin wax composites were measured during cooling under nitrogen atmosphere. DSC measurements were performed at 10 °C/min heating and cooling rates for temperature range from room temperature to 90 °C. Scanning electron microscopy (SEM) was carried out using QUANTA FEG250 SEM instrument (FEI, Hillsbor, OR, USA) to determine the particle size.

## 3. Results and Discussion

### 3.1. Morphology

**Figure 2:**
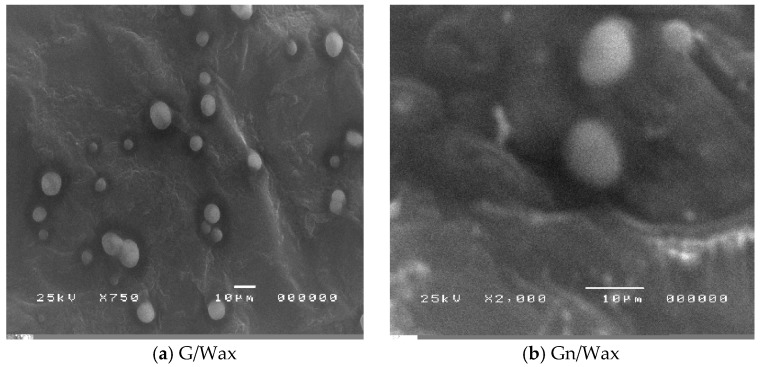
Scanning Election microscopy of paraffin wax hosting: (**a**) graphite dispersed in toluene; and (**b**) graphene.

Scanning electron microscopy (SEM) confirms further homogenity of the paraffin composite samples (Figure 2). The average particle size is about 6.2 and 8.4 µm for graphite and graphene, respectively, in paraffin composites.

### 3.2. DSC of Paraffin Wax Composites

The influence of graphite and graphene addition on the thermal properties including the melting temperatures and latent heat of fusion were analyzed from the DSC measurement. The DSC of pure paraffin wax is a reference to evaluate the thermal properties changes depending on the paraffin graphite composite ratio.

**Figure 3:**
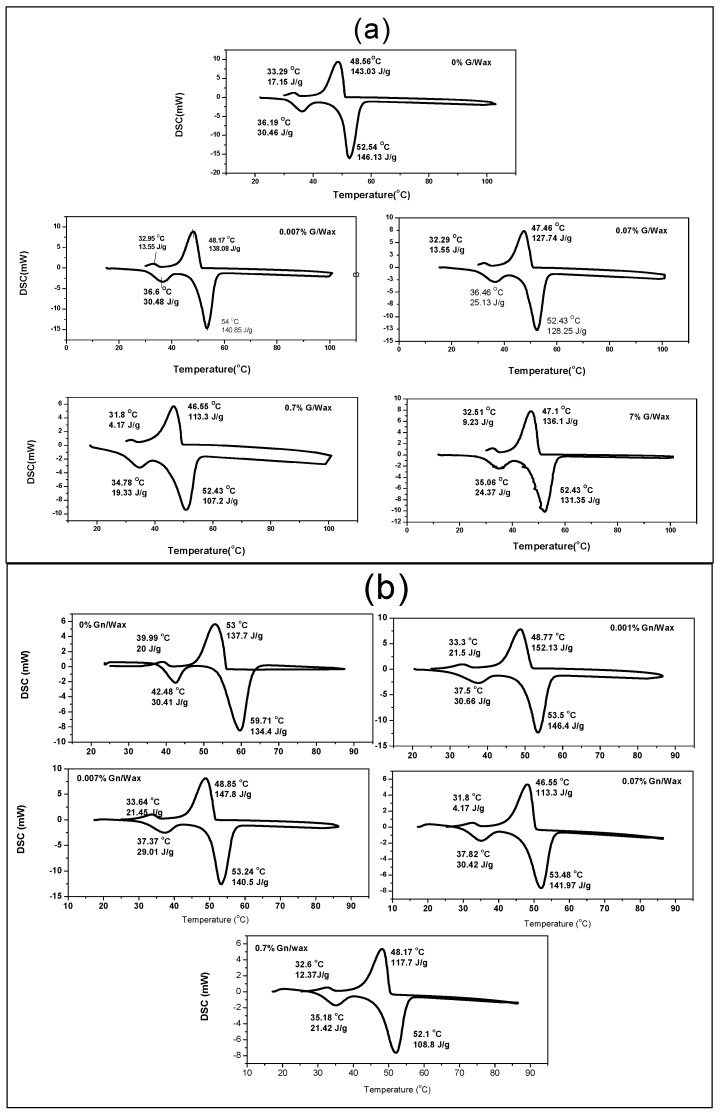
DSC of Paraffin wax composites of different concentrations: (**a**) G/wax; and (**b**) Gn/wax. DSC curves showing solid-solid transitions and solid–liquid transitions during heating and cooling phases; the phase change temperatures and latent heat of transitions are indicated.

It can be seen from DSC curves (Figure 3) that the heating curves represent the absorbed energy by the material in endothermic phase transition while cooling curves represent the expelled energy by the material in exothermic phase transition. For pure paraffin wax, there are two endothermic peaks in the DSC curves (Figure 3a): the main peak corresponds to solid–liquid phase change T_2_ of the paraffin at about 52.54 °C and the minor peak represents solid–solid phase transition T_1_ of the paraffin wax at about 36.19 °C. These peaks match the pure paraffin wax peaks in other reports [4,32]. There are also two endothermic peaks for paraffin wax graphite composites: the solid–liquid peak T_2_ fluctuates around 52 °C, while the solid–solid T_1_ transition peak is altered from 36 to 34 °C. The thermal properties of the paraffin wax-graphite composites are very close that of pure paraffin wax. This is because there are no chemical interactions between paraffin wax and graphite through preparation of the samples. Zhang et al. [33,34] observed the same behavior for expanded graphite-paraffin wax composite.

For DSC of Paraffin wax/Gn (Figure 3b), during the heating phase, T_1_ is decreased from 42 to 35 °C and T_2_ is decreased from 59 to 52 °C as the concentration of the graphene increases. In the cooling phase, there is a noticeable decrease from 53 to 48 °C and from 40 to 32 °C, respectively, as the concentration of the graphene increases. There may be chemical reactions between the graphene and paraffin wax, which needs further measurements, such as X-ray diffraction (XRD), which will be completed in further work.

**Figure 4:**
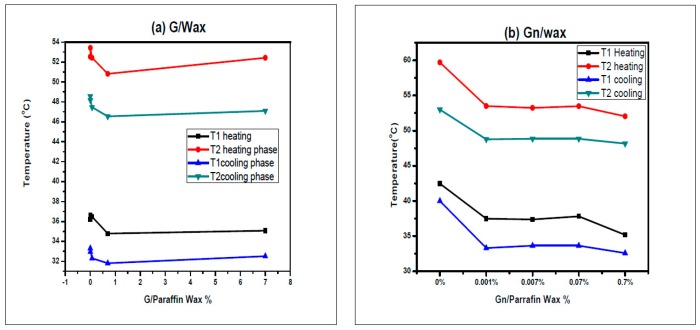
The solid-solid transition temperature, the solid liquid transition temperature during heating and cooling phase for: (**a**) G/paraffin; and (**b**) Gn/Paraffin.

There is no big fluctuation in the melting and solidification temperatures for graphite-paraffin wax composites, while there is a slight decrease for the melting and solidification temperatures for grapheme-wax composites (Figure 4). Fan et al. [35] recorded a similar variation of the melting and solidification temperatures. They observed no clear trend between the melting temperature variation and the loading of carbon nanofillers. They attributed these variations to the filler induced alignment of paraffin molecules surrounding the carbon, which alters the local steric hindrance. Xiang et al. [3] present DSC results that show the latent heat of paraffin nanocomposites were not degraded by adding exfoliated graphite nanoplatelets, while Zhang et al. [33] found that the phase change temperature were very close to that of paraffin while the latent heat of the composite was equal to the latent heat of the paraffin multiplied by its mass fraction [9].

**Figure 5:**
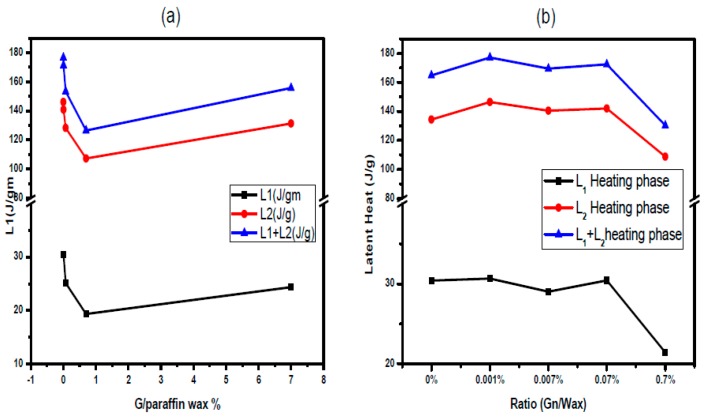
Latent heat during heating phase for solid–solid transition L_1_ and solid liquid transition L_2_ and their sum L_1_ + L_2_ for: (**a**) G/wax; and (**b**) Gn/wax.

The latent heats for solid-solid transition L_1_ and solid liquid transition L_2_ are calculated from DSC spectra as the total area under the transition peak of the composite by numerical integration. The latent heat is decreased for paraffin wax composites as the host ratio increases (Figure 5). The latent heat is nearly equivalent to the calculated one based on the value of the latent heat of pure paraffin wax multiplied by (one mass ratio of the host). However, there are discrepancies for higher mass ratios for graphite percentages 0.7% and 7% and for Gn percentages 0.07% and 0.7%. Li et al. [36] observed a decrease of the latent heat of decosane-spongy graphene composite with the graphene addition.

Specific heat capacity of composites is calculated with Equation (1) using the DSC data of the composites [37].

C_p_ = E·Q/R·M, (1)

where C_p_ is the specific heat capacity, Q is the heat flow, R is the heating rate (T/t), M is the mass of sample, and E is the DSC calibration factor.

DSC calibration factor E is calculated knowing the specific heat capacity of paraffin wax (2926 J/Kg·°C), heat flow, heating rate (T/t) (10 °C/min) and mass of the sample. The value of DSC calibration factor is used to find specific heat capacity of other different concentration of graphite or graphene.

**Figure 6:**
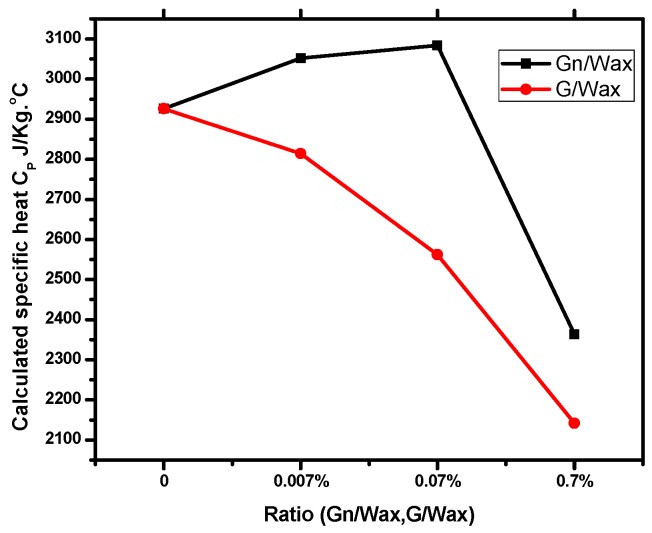
Calculated specific heat capacity of solid-liquid phase transition of composites during heating.

The calculated specific heat capacity of paraffin wax composites is decreased as the host concentration (Gn or G) is increased (Figure 6). Thus, a smaller quantity of heat is needed for carbon fillers paraffin composites to raise their temperatures. Both carbon fillers (G and Gn) have great effects in reducing the specific heat capacity of paraffin wax.

### 3.3. Thermo-Optical Switching of Paraffin Wax Spot Composites under Electrical Heating

**Figure 7:**
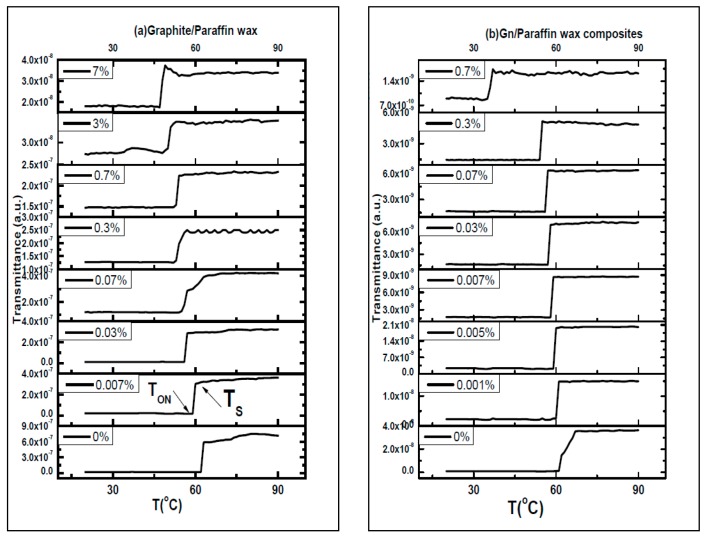
Thermo-optical switching TOS transmission of spots paraffin wax composite spots hosting: (**a**) graphite; and (**b**) graphene versus temperature during heating phase. The switching temperature (T_ON_) and saturation temperature (T_S_) are indicated.

Thermo-optical switching transmission is measured under electric heating, as shown in Figure 7. At room temperature, the composites are opaque to light transmission because paraffin wax composite are in solid state. By raising the temperature, the transparency of the samples increases, and the transmission of diode laser under heating is increased.

T_ON_ is the temperature at which the temperature starts to increase under heating and T_S_ is the temperature at which the transmission saturates. The switching temperature (T_ON_) and saturation temperature (T_S_) of paraffin wax-spot composites are deduced from the switching curves shown in Figure 7.

Paraffin wax-graphite composites spots dispersed in toluene switch at 62, 59, 56, 54.9, 52.8, 52.8, 49 and 47 °C and saturate at 63, 59.8, 57, 57, 56.4, 54.2, 51 and 48.9 °C as graphite to paraffin wax ratios increases during heating phase, while spots of paraffin wax-graphene composites switching temperature (T_ON_) are 60.6, 60, 59.1, 58.5, 57, 56, 53 and 34 °C and saturation temperature (T_S_) are 66, 61.2, 60.5, 59.2, 58.5, 57.4, 55 and 37.4 °C as the graphene to paraffin wax ratios are increased during heating, as shown in Figure 7.

**Figure 8:**
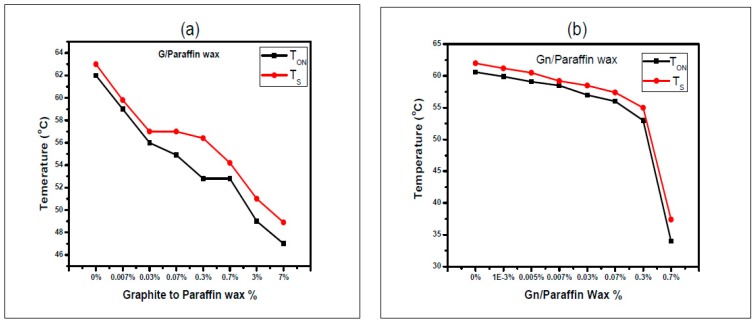
The switching Temperature T_ON_ and the saturation Temperature T_S_ versus: (**a**) G/Wax; and (**b**) Gn/Wax, during heating phase.

The relation between T_ON_ and T_S_ as a function of the composite ratio hosting graphite is almost linear, as shown in Figure 8, The most remarkable values of the switching temperature are at the highest concentration of graphene, and sharp decreases of T_ON_ and T_S_ are observed. The difference between the switching temperature T_ON_ and the saturation temperature T_S_ is less for graphene/wax composites than that of graphite/wax composites. These findings confirm lowering the temperature rise due to the extreme thermal conductivity of the graphene over that of graphite.

**Figure 9:**
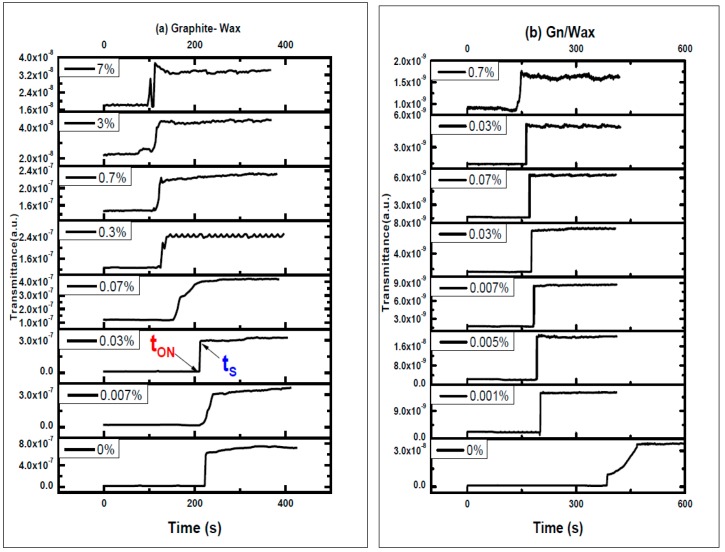
TOS of: (**a**) paraffin wax-graphite composites spots; and (**b**) Gn/Paraffin wax versus time during heating phase. Switching time t_ON_ and saturation time t_S_ are indicated.

Figure 9 shows TOS of paraffin wax composite spots versus time during heating phase. As the percent of graphite and graphene in the paraffin composites increases, there is a slight decrease in both the switching time t_ON_ and the saturation time t_S_ (t_ON_ is the time where the transmission starts to increase and t_S_ is the time where the transmission reaches its maximum value). Switching times (t_ON_) of paraffin wax-graphite composite spots dispersed in toluene are 223, 216, 211, 155, 122, 117, 103 and 96 s and saturation times (t_S_) are 225, 241, 213.7, 167, 139, 125, 117 and 115 s, as graphite to paraffin wax percent increases during heating, as shown in Figure 9.

**Figure 10:**
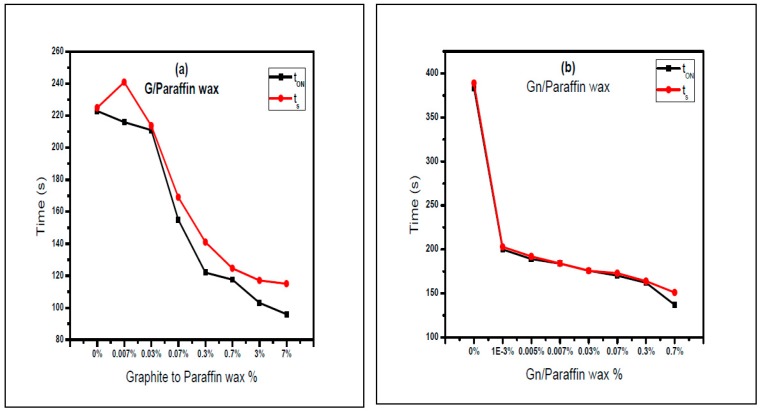
t_ON_ and t_S_ versus (**a**) G/Wax; (**b**) Gn/Wax under electric heating.

The switching ON time (t_ON_) and saturation time (t_S_) of paraffin wax-graphene spot composites are 381.4, 200, 189, 183, 177.8, 170.2, 162 and 137 s and 389, 203, 192, 185, 180.3, 173, 164 and 151 s. The switching ON time (t_ON_) and saturation time (t_S_) of paraffin wax-graphene spot composites are decreased as graphene to paraffin wax ratios increase during heating, as shown in Figure 10. The most striking result to emerge from the data is that the rising time Δt = (t_S_ − t_ON_) was enhanced for Gn/Wax spots.

Thermo-optical parameters of paraffin wax are enhanced with addition of graphene and graphite. A decrease in the switching and saturation temperatures and their corresponding switching on time and saturation time t_S_ are observed. These lead to fast switching as compared to paraffin wax, as the content of carbon fillers in paraffin wax are increased; consequently, the quantity of heat transferred by convection through paraffin wax-carbon filler composites are increased in comparison to that transferred through pure paraffin wax. Once the paraffin wax is completely melted, convection plays a role in the heat transfer. The time necessary for melting is reduced by the role of the natural convection of the thermal conductive fillers. As interpreted by Yu et al. [38], the better performance of Gn is primarily attributed to its planar structure, which lowers the geometric contribution of phonon mismatch, leading to less phonon scattering at the interfaces between the filler and the matrix material. Consequently, the enhancement of TOS is not only due to high thermal conductive carbon fillers but is also due to the filler-induced alignment of the paraffin molecules that inherently enhances the thermal properties of the paraffin wax.

The observations of TOS transmission cooling curves correspond to those from heating curves. TOS transmission cooling curves are presented in Appendix A.

### 3.4. Thermo-Optical Switching of Paraffin Wax Composites under Nd:YAG Laser Heating

TOS of paraffin wax composite spots in the center of copper capillary tube are studied. Using copper capillary tube is mandatory for the measurements because glass capillary tube broke due to the high power of Nd:YAG laser, while other forms such as Teflon and stainless steel do not match the correct measurements. Copper has larger thermal conductivity and smaller heat capacity than glass, which lead to efficient and fast heat transfer of laser pulses to the paraffin wax composite spots.

When the Nd:YAG laser is off, the sample is opaque. The light coming from diode laser through optical fiber is blocked. Nd:YAG laser (1064 nm) with high power laser pulses (110 mJ) is directed normally to the capillary copper tube as a heating source; the heat is transferred from laser beam through the copper wall of the capillary to melt the wax spot. The light, through optical fiber, is passed through the melted spot. Transmission is recorded as a function of time via optical power meter. Recording temperature variation was difficult under laser heating. An estimation of the temperature under laser heating is done in Appendix B. Two series of paraffin wax spot composites using graphite/graphene are studied under laser heating.

**Figure 11:**
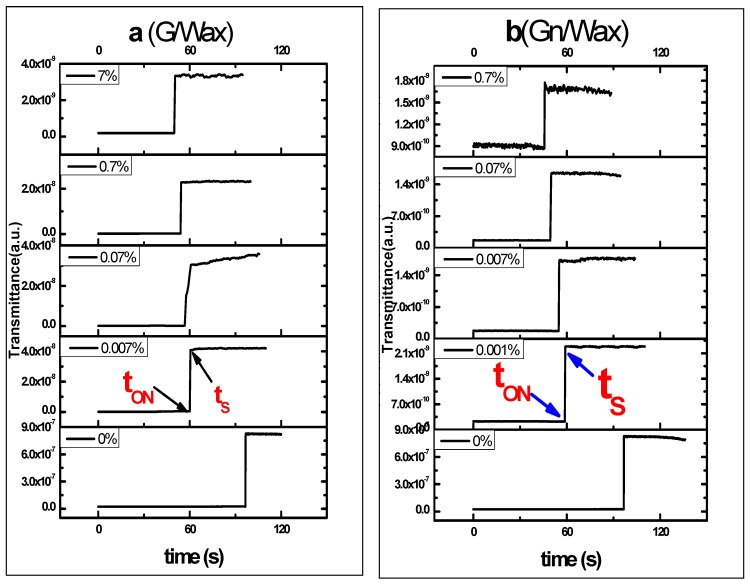
TOS of paraffin wax spots hosting: (**a**) graphite; and (**b**) graphene composites versus time for different concentration of host under laser heating. The switching ON time (t_ON_) and saturation time (t_S_) are marked on the figure.

The switching ON time (t_ON_) and saturation time (t_S_) of paraffin wax-graphene composite spots during heating are deduced from the switching curves shown in Figure 11.

By using Nd:YAG laser as a heating source, paraffin wax-graphite spot composites dispersed in toluene switching on times T_ON_ are 95.6, 59.5, 56, 54 and 49.2 s. The saturation time t_S_ are 96.7, 60.5, 60.8, 54.4 and 50.4 s with graphite to paraffin wax percentages 0%, 0.007%, 0.07%, 0.7% and 7%, respectively, during heating phase, as shown in Figure 11.

The switching ON time (t_ON_) of paraffin wax-graphene composite spots under laser heating are 94, 58.5, 54, 49.5 and 45.6 s. Saturation times (t_S_) are 96.7, 59.1, 55.2, 50 and 45.8 s with graphene to wax percentages 0%, 0.001%, 0.007%, 0.07% and 0.7%, respectively, during heating phase.

**Figure 12:**
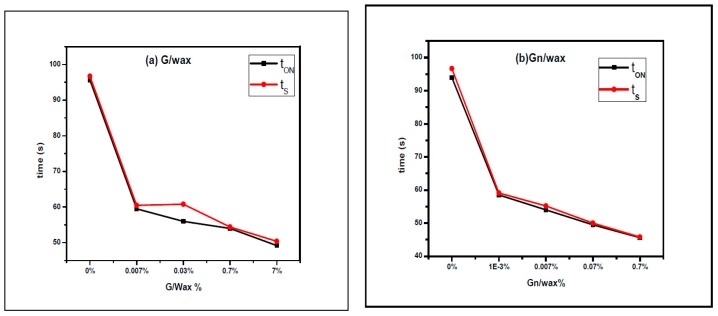
The measured switching ON time (t_ON_) and the measured saturation time (t_S_) of paraffin wax-graphite composites hosting: (**a**) graphite; and (**b**) graphene using Nd:YAG laser as a heating source.

The switching on time (t_ON_) and measured saturation time (t_S_) of paraffin wax-graphene spot composites under laser heating are shown in Figure 12. During heating, the total laser exposure time for melting the sample completely is decreased as the carbon filler ratio increases (G and Gn). The gap between switching and saturation times (rise time Δt) reaches 0.2 and 0.4 s for 0.7% Gn/wax and 0.7% G/wax, respectively. These results highlight using paraffin wax with best concentrations of G/Gn under laser exposure in thermo-optical switching applications.

Thermo-optical switching times are summarized in Table 1 for 0%, 0.007%, 0.07% and 0.7% spots paraffin wax composite under electric and laser heating. Furthermore, a comparison to Ref. [29] of higher mass of paraffin wax is included in the table. The results in Table 1 confirm decreasing the switching times for small mass of paraffin wax composite spots.

By comparing t_ON_ and t_S_ for graphite-wax spots using electric heating and laser heating for the above percentages 0%, 0.007%, 0.07% and 0.7%, a much decrease in t_ON_ and t_S_ are observed under laser heating. An enhancement factor is calculated by;

(2) $$\eta \;t_{\mathrm{ON}}=|\frac{t_{ON}\;under\;laser\;heating-t_{ON}\;under\;electric\;heating}{t_{ON}\;under\;elctric\;heating}|\times 100\%$$

(t_ON_ or t_S_ under electric heating) for switching and saturation times, respectively.

Equation (2) represents the difference between the switching times of wax spots hosting carbon fillers under laser heating and electric heating divided by the switching time for the same filler concentration under electric heating. Similarly, the time enhancement factor for t_S_ is η t_S_.

For G/wax, the enhancements for t_ON_ were 57%, 72.5%, 63.9%, and 53.9%, while the enhancements for t_S_ for were 75.4%, 70.6%, 70.9%, and 66.7% for 0%, 0.007%, 0.07% and 0.7%, respectively.

For Gn/wax, the enhancements for t_ON_ were 75.4%, 70.5%, 70.9%, and 66.7%, while the enhancements for t_S_ for were 75.1%, 70%, 71.1%, and 69.7% for the above-mentioned ratios.

Significantly, In addition to thermal heating effect of laser which transfers heat effectively in a shorter time of few milliseconds, enhancements of switching times of Gn/wax are greater for those of G/wax due to the extreme thermal conductivity of Gn.

In this work, the TOS based on the transmission change undergoing phase change material (PCM) is investigated, the switching efficiency are enhanced by adding high thermal conductive fillers to PCM.

In the literature, thermo-optic effect depends on the refractive index variation of the material due to temperature variation of the material. The most used materials to fabricate TOS are polymers, silica and silicon. Conventional thermo-optic waveguide switches are usually based on the Y-branch, Mach–Zehnder interferometer (MZI) and Total Internal Reflection (TIR). Y-branch digital optical switch (Y-branch DOS) principle lies on refractive index exchange of light input into the “base” or trunk of the Y and the output branches. In Mach–Zehnder interferometer, heating one arm of the interferometer causes its refractive index to change; hence, a variation of the optical path between the two interferometer arms is experienced. Consequently, a phase difference between the light beams may be constructive or destructive interference and selection of the output can be maximized or minimized. While thermo optic switch based on TIR depends on changing the path of light passing from a prism; with increasing the prism temperature, the refractive index of the prism increases. Consequently, the critical angle decreases and becomes less than the incident angle; hence, the incident light is totally reflected in other output [1,2].

Y branch TOS and MZI TOS configurations have complementary advantages and disadvantages. Y branch structure has the advantages of larger operation bandwidth and medium device size, but has the disadvantages of larger power consumption. MZI has the advantages of low power consumption. However, it has the disadvantages of narrower operation bandwidth and larger device size [39].

Table 2 summarizes some optical switching configurations based on different materials. The switching time is reported, Optical switching speed ranges from microsecond up to millisecond depending on the switch design. The main disadvantage of our investigated thermo-optic switch is the slow switching in comparison to other TOS configurations. There are two ways to decrease the switching time: the first is by decreasing the mass of paraffin wax spot, and the second is by enhancing the thermal properties of paraffin wax.

Our thermo-optical switching design can be useful for cost-effective switching designs but it suffers low switching time. The investigated technique is simple, low cost and low alignment compared to other TOS high complex structures. Our work clearly has some limitations. Nevertheless, it could be a springboard to using phase change materials as thermo-optic switch.

## 4. Conclusions

This paper has given an account of thermo-optical switching of paraffin wax spots hosting carbon fillers (G/Gn). The morphology of paraffin composites was determined via SEM. The images show better homogeneity for the prepared paraffin composites. DSC measurements show phase change temperatures of solid-solid and solid-liquid transitions of paraffin wax composites. The latent heat and specific heat capacity are calculated based on DSC data. We have confirmed that a small quantity of heat is needed to raise the composite temperature from specific heat capacity calculations.

The evidence from this study points towards the idea of enhancing thermo-optic switching of paraffin wax spot composites hosting G/Gn under electric heating as well as Nd:YAG laser heating. We have obtained comprehensive results demonstrating the switching time t_ON_ and saturation time t_S_ are enhanced for paraffin wax composites under laser heating. The heating by Nd:YAG laser led to faster switching than using continuous heating (electric heater). The decreases in rise time (t_S_ − t_ON_) of the switches were up to 0.2 and 0.4 s for paraffin wax spots hosting graphene and graphite, respectively. The enhancement of lowering the switching parameters is due to the high thermal effect of high power laser pulses. The upshots of these measurements support using paraffin wax (PCM) hosting graphite or graphene as thermo optical sensing material for Thermo-optical Switching (TOS) device applications.

## Figures and Tables

**Table 1:** Thermo-optical switching times of paraffin wax composite spots under electric heating, laser heating in comparison to Ref. [29]. t_ON_: the switching on time; t_S_: the saturation time; ∆t: rise time.

**(a)**										
**G-Paraffin Wax**										
**G/Wax%**	**Spot under Electric Heating**			**Spot under Laser Heating**			**η t_ON_**	**η t_S_**	**Said et al, Ref. [29]**	
	**t_ON_/s**	**t_S_/s**	**∆t/s**	**t_ON_/s**	**t_S_/s**	**∆t/s**			**t_ON_/s**	**t_S_/s**
0	223	225	2	95.6	96.7	1.1	57.1	57	480.3	501
0.007	216	241	25	59.5	60.5	1	72.5	74.9	347.8	352
0.07	155	167	12	56	60.8	4.8	63.9	63.6	490	497
0.7	117	125	8	54	54.4	0.4	53.9	56.5	592	597
**(b)**										
**Gn-Paraffin Wax**										
**Gn/Wax%**	**Spot under Electric Heating**			**Spot under Laser Heating**			**η t_ON_**	**η t_S_**	**Ref. [29]**	
	**t_ON_/s**	**t_S_/s**	**∆t/s**	**t_ON_/s**	**t_S_/s**	**∆t/s**			**t_ON_/s**	**t_S_/s**
0	381.4	389	7.6	94	96.7	2.7	75.4	75.1	488.1	495.1
0.007	183	185	2	54	55.2	1.2	70.5	70	409	430
0.07	170.2	173	2.8	49.5	50	0.5	70.9	71.1	258.6	267.6
0.7	137	151	14	45.6	45.8	0.2	66.7	69.7	149.9	156.1

**Table 2:** Some optical switching configuration based on different materials, Digital thermo optic switch: DOS; Thermo-optic switch: TOS; total internal reflection: TIR; and Mach–Zehnder interferometer: MZI.

Configuration	Material	Switch Time	Ref.	Year
DOS	polymer	10 ms	Noh et al. [40]	2006
TOS based on TIR	polymer	3 ms	Han et al. [41]	2012
Y branch	chiral azobenzene-containing polyurethane (CACPU)	12 ms	Ye et al. [42]	2013
MZI	chiral azobenzene-containing polyurethane (CACPU)	2 ms	Ye et al. [42]	2013
TOS based on TIR	Polyimide waveguide	Not reported	Yang et al. [43]	2002
Liquid-optical switch	Conductive liquids	132 ms	Liu et al. [44]	2015
IR switch	glycerol droplet	200 ms	Ren et al. [45]	2012
TOS (MZI)	Silicon on insulator (SOI)	141 µs	Sun et al. [46]	2010

**Figure A1:**
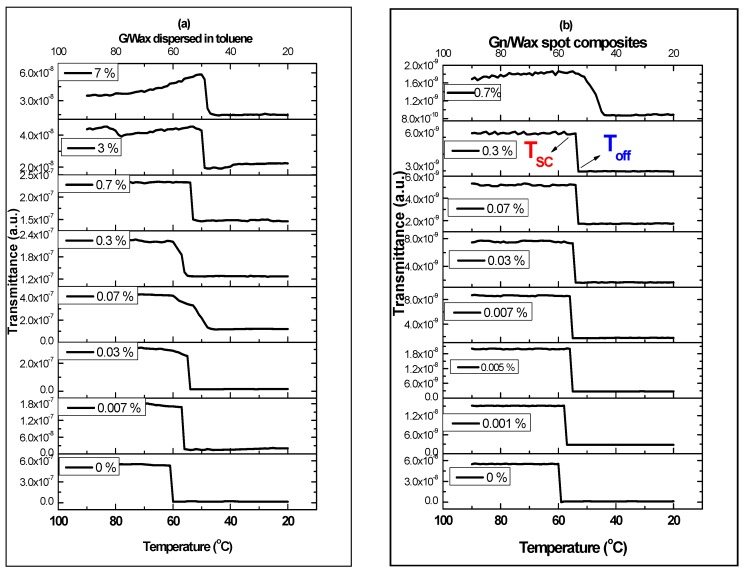
TOS cooling curves of paraffin wax-spot composites hosting: (**a**) graphite dispersed in toluene; and (**b**) graphene versus temperature. T_sc_ and T_off_ are marked in the figure.

**Figure A2:**
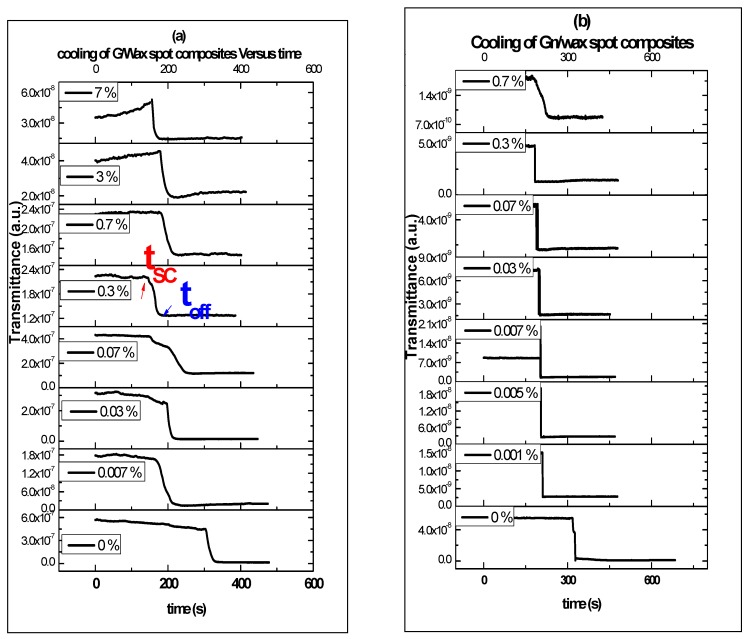
TOS cooling curves versus time of paraffin wax spot composites hosting: (**a**) G; and (**b**) Gn.

**Figure A3:**
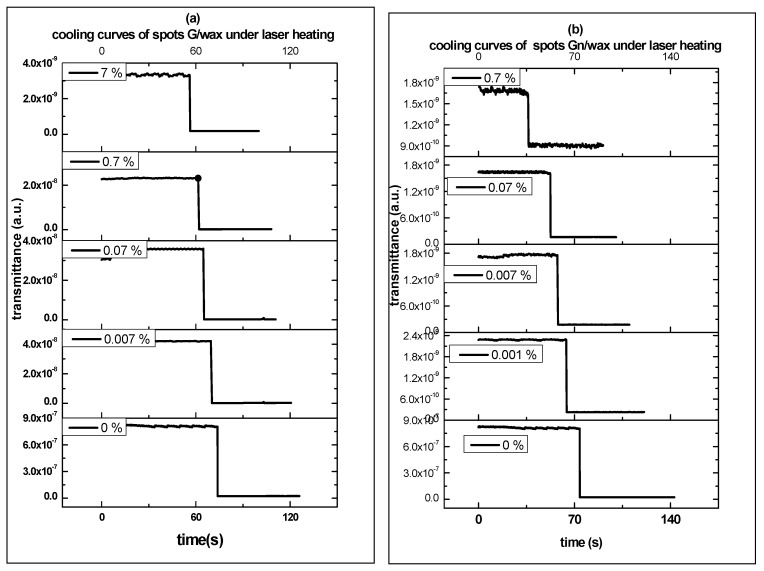
TOS cooling curves after laser heating of paraffin wax spot composites hosting: (**a**) G; and (**b**) Gn.

**Figure A4:**
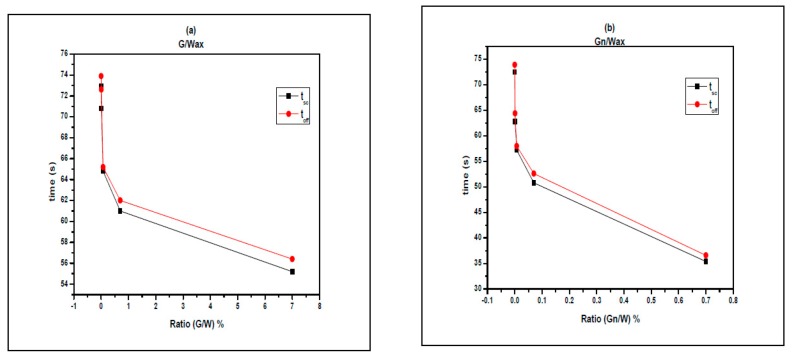
The measured times t_sc_ and t_off_ of paraffin wax-graphite spot composites hosting: (**a**) graphite; and (**b**) graphene during cooling after using Nd:YAG laser.

**Figure A5:**
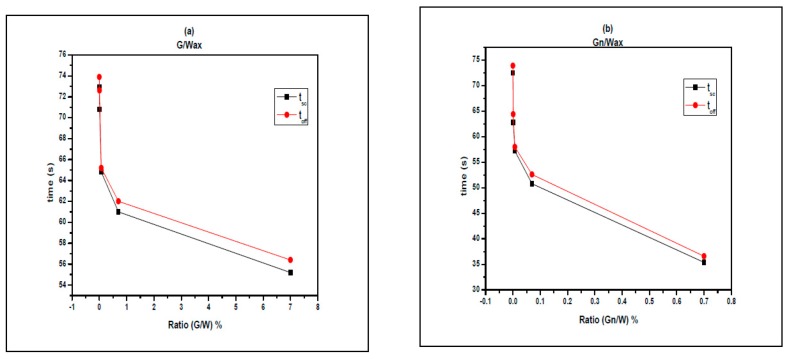
Estimated melting temperature (T_m_) of: (**a**) graphite/wax (%); and (**b**) graphene/wax (%) during heating by using Nd:YAG laser as a heating source.

## Appendix A

### Appendix A.1. TOS Cooling Curves of Paraffin Wax Spot Composites under Electric Heating

As shown in Figure A1, T_sc_ is the temperature at which the transmitted intensity starts to decrease while T_off_ is the temperature at which the transmitted intensity is blocked and the composite returns in the solid phase. There are fluctuations of T_sc_ and T_off_ in the cooling phase as the concentration of Graphite increases, while the Gn shows a greater decrease of T_sc_ and T_off_ as its percent increases in the composite.

The T_sc_ of spots paraffin wax-graphite composites dispersed in toluene were at 62, 57, 55, 60, 52, 52, 49 and 47 °C and T_off_ were at 60, 56, 54, 48, 60, 53, 48 and 46 °C as graphite to paraffin wax ratios increase, respectively, during cooling phase.

T_sc_ and T_off_ of spots paraffin wax-graphene composites, which were at 58, 56, 55, 55, 54, 53, 53 and 44 °C and 60, 58, 56, 56, 55, 54, 54 and 52 °C, respectively, are decreased as graphene to paraffin wax ratios are increased during cooling.

t_sc_ is the time at which the transmitted intensity starts to decrease at corresponding temperature T_sc_ while t_off_ is the time at which the transmitted intensity is blocked at corresponding temperature T_off_ and the composite returns to the solid phase.

t_sc_ and t_off_ of spots paraffin wax-graphite composites dispersed in toluene were 308.27, 163.04, 197.38, 151.4, 146.47, 181.86, 180.38 and 158.19 s and 328.31, 219.86, 214.24, 252.4, 178.20, 218.01, 204.06 and 173.5 s as graphite concentrations to paraffin wax ratios increases, respectively, during cooling phase.

The t_sc_ and t_off_ of spots paraffin wax-graphene composites were 315.37, 209.84, 203.12, 201.47, 197.38, 189.80, 186.50 and 180.5 s and 327.5, 214.79, 204.77, 206.55, 203.42, 193.10, 183.08 and 234.12 s, respectively, for the mentioned concentrations (Figure A2).

### Appendix A.2. TOS Cooling Phases of Paraffin Wax Spot Composites after Exposure to Nd:YAG Laser Pulses

By switching off Nd:YAG pulsed laser, the times t_SC_ and toff of spots paraffin wax-graphite composites dispersed in toluene are 72.9, 70.8, 64.8, 61 and 55.2 s and 73.9, 72.6, 65.2, 62 and 56.4 s for graphite to paraffin wax percentages 0, 0.007, 0.07, 0.7 and 7, respectively, during cooling phase, as shown in Figure A3.

t_SC_ and t_off_ of spots paraffin wax-graphene composite were 72.5, 62.8, 57.2, 50.8 and 35.4 s and 73.9, 64.4, 58, 52.6 and 36.6 s for graphene to paraffin wax percentages 0, 0.001, 0.007, 0.07 and 0.7, respectively, during cooling, as shown in Figure A3.

The cooling times t_SC_ and t_off_ are sharply decreased as the concentrations of the carbon filler are increased (Figure A4). The carbon fillers enhance the switching times in the heating and cooling phases.

## Appendix B

### Estimation of Melting Temperature T_m_ Using TOS Measurements under Laser Heating

We assumed that the sample under study is exposed to number of laser pulses N:

N = FRR × t_ON_ + t_s_/2
(A1)

where FRR is frequency Repetition Rate (10 Hz), t_ON_ is the switching ON time where the transmitted intensity starts to increase, and t_s_ is the saturation time where the transmitted intensity reaches to maximum. We assumed the melting temperature T_m_ at time t_ON_ + t_s_/2, assuming that paraffin wax is melted exactly.

The incident energy after N laser pulses on the center of the sample can be calculated from the following equation:

Q = energy pulse × N
(A2)

The loss of energy is estimated to be from reflection and mismatching between laser beam area and copper capillary tube area Q loss.

The effective energy Q _eff_. transferred to the composite can be calculated by:

Q _eff_. = Q − Q loss
(A3)

Melting temperature can be calculated using TOS transmission measurements under pulsed laser heating:

Q _eff_. = m_1_ × c_1_ × ΔT_1_ + m_2_ × c_2_ × ΔT_2_ + m_2_L
(A4)

where

- m_1_ is the mass of copper sheet and m_2_ are the mass paraffin wax-graphite or graphene dot composites.
- C_1_ and C_2_ are specific heat capacity of copper sheet and paraffin wax-graphite or graphene dot composites, respectively.
- L is the latent heat of paraffin wax composites calculated from DSC measurements.
- ΔT = T_m_ − T_i_ where T_i_ = Troom = 30 °C.
- Neglect the second term m_2_ × c_2_ × ΔT_2_ due to the small weight of mixture (wax + graphite or graphene).
- The melting temperature is estimated as a midpoint between t_ON_ and t_S_.
- The effective area used in calculation as a ratio of laser beam area to surface area of the copper capillary tube.
- The calculated value of the specific heat is from DSC measurement for paraffin wax-graphite or graphene ratio.

Figure A5 shows the estimated melting temperature of paraffin wax spot composites. T_m_ is decreased as the carbon filler increases in the composite under laser exposure, which is attributed to the high thermal conductivities of the G and Gn.
